# Association of blood trihalomethane concentrations with diabetes mellitus in older adults in the US: a cross-sectional study of NHANES 2013–2018

**DOI:** 10.3389/fendo.2024.1401131

**Published:** 2024-07-08

**Authors:** Tuotuo Chen, Haiqing He, Wei Tang, Ziyi Liu, Hongliang Zhang

**Affiliations:** ^1^ Department of Emergency Medicine, The Second Xiangya Hospital, Central South University, Changsha, Hunan, China; ^2^ Emergency and Difficult Diseases Institute of Central South University, Changsha, Hunan, China; ^3^ Department of Urology, The Second Xiangya Hospital, Central South University, Changsha, Hunan, China; ^4^ Department of Nephrology, The Second Xiangya Hospital, Central South University, Changsha, Hunan, China

**Keywords:** trihalomethanes, diabetes mellitus, disinfection by-products, NHANES, older adults in the US

## Abstract

**Background:**

Previous studies have demonstrated that there is a correlation between trihalomethanes and disease progression, such as allergic diseases. As we know, only few studies focused on the relationship between trihalomethanes and metabolic diseases, such as diabetes mellitus.

**Objective:**

The aim of this study was to further explore the associations between blood trihalomethane concentrations and diabetes mellitus in older adults in the US.

**Methods:**

Data were collected from the National Health and Nutrition Examination Study (NHANES) database in the survey cycle during 2013 to 2018, including 2,511 older adults in the US whose blood trihalomethane concentrations were measured, involving chloroform (TCM) and brominated trihalomethanes (Br-THMs). Br-THMs include bromodichloromethane (BDCM), dibromochloromethane (DBCM), and bromoform (TBM). Meanwhile, the concentration of total trihalomethanes (TTHMs) was also measured later. A multivariate logistic regression and restricted cubic spline were used to examine the relationship between blood THMs and diabetes mellitus. Meanwhile, we performed a subgroup analysis, which aims to explore the stability of this relationship in different subgroups. In order to further consider the impact of various disinfection by-products on diabetes, we also used weighted quantile sum (WQS). To explore the correlation in trihalomethanes, we plot a correlation heatmap.

**Results:**

Adjusting for potential confounders, we found that there was a significant negative association between chloroform and diabetes mellitus [Model 1 (adjusted for covariates including age, sex, and race, OR = 0.71; 95% CI: 0.50–1.02; *p* = 0.068; *p* for trend = 0.094); Model 2 (adjusted for all covariates, OR = 0.68; 95% CI: 0.48–0.96; *p* = 0.029; *p* for trend = 0.061)]. In the bromodichloromethane, we reached a conclusion that is similar to TCM [Model 1 (adjusted for covariates including age, sex, and race, OR = 0.54; 95% CI: 0.35–0.82; *p* = 0.005; *p* for trend = 0.002); Model 2 (adjusted for all covariates, OR = 0.54; 95% CI: 0.35–0.82; *p* = 0.003; *p* for trend = 0.002)]. Meanwhile, the restricted cubic spline curve also further confirms this result (*p* overall = 0.0027; *p* overall< 0.001). Based on the analysis in the subgroups, we found that the value *p* for interaction in the majority of subgroups is higher than 0.1. Trihalomethanes and diabetes were inversely associated, and in the WQS, chloroform and bromodichloromethane were found to be the major contributors to this relationship. In the correlation analysis, we found that most trihalomethanes have a weak correlation, except for TBM and TCM with a strong correlation.

**Conclusion:**

Our results in this study showed that blood chloroform, bromodichloromethane concentrations, and diabetes mellitus in older adults in the US are negatively correlated, suggesting that chloroform and bromodichloromethane can be protective factors for diabetes.

## 1 Introduction

Diabetes mellitus is a systemic disease, whose clinical manifestations usually include polydipsia, polyuria, overeating, and weight loss. Although the etiology of diabetes mellitus is quite complicated, some studies indicated that it can be attributed to lack of insulin secretion or insulin resistance or both (1). Long-term hyperglycemia can lead to damage to the function of our systemic organs, particularly the eyes, kidneys, nerves, heart, and blood vessels (2–7). Some studies showed that the population of patients with diabetes in recent years is increasing gradually (8). As the global population ages, the prevalence of diabetes is evidently increasing in older adults (9). It is expected that the economic burden of diabetes will increase in the next few decades. Therefore, it is necessary to focus on the causative and protective factors of diabetes in the older adult population.

Chlorine disinfection of public water supplies remains one of the main means to control the microbial contamination around the world. When disinfectants (such as hypochlorous acid and calcium hypochlorite) react with substances in the water, more than hundreds of water disinfection by-products (DBPs) are produced (10). Humans are exposed to DBPs when people use water for some daily activities (e.g., swimming, drinking, and bathing). Therefore, residual contaminants formed during water disinfection may have adverse health effects (11). Trihalomethanes are one of the most common DBPs and the humans are most easily exposed to this DBP. Several studies have linked trihalomethanes to some allergic diseases, such as asthma (12, 13). In addition, on the relationship between THMs and diabetes mellitus, some scholars have different opinions. On the one hand, in diabetic rats with diet and alloxan-induced diabetes, the chloroform fraction of plants (such as *Anthocleista vogelii* Planch root bark) has antidiabetic effects (14). On the other hand, it has been shown that brominated trihalomethanes (Br-THMs) are a risk factor for diabetes, contributing to related diabetic events through leptin and liver damage (15, 16). The barriers to scholars’ views give us enough motivation to further explore the relationship between trihalomethanes in human blood and diabetes mellitus. The determination of trihalomethane concentrations requires consideration of several factors, and other exposure pathways may have been overlooked in previous determinations of THMs in tap water. Blood trihalomethane concentrations represent a more integrated measurement of multiple exposure routes and sources (17). Steady-state blood concentrations generally depend on the frequency of exposure events. The time spent swimming in a chlorinated swimming pool is generally positively correlated with blood trihalomethane concentrations. Showering, washing dishes by hand, and ingestion of hot beverages made with tap water are associated with higher blood THMs (18). In addition, there are no research evaluating the relationship between blood trihalomethanes and diabetes mellitus in older adults. The immune system of older adults may be more fragile than the rest of the population. Therefore, to further explore the association of trihalomethanes with diabetes mellitus, we used the National Health and Nutrition Examination Study (NHANES) database and selected older adults as our study group.

## 2 Methods

### 2.1 Study population

**Figure 1:**
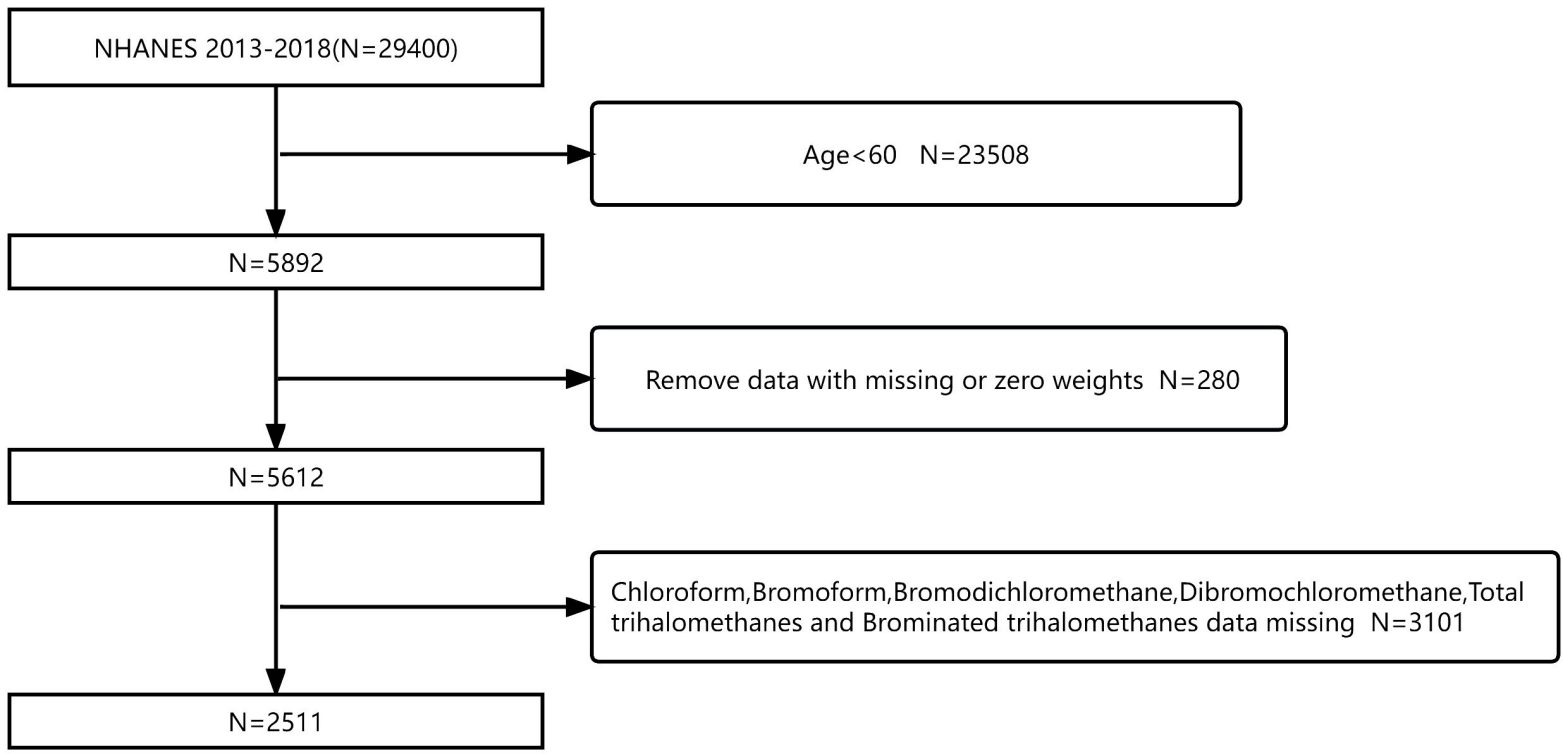
Flowchart of participant screening based on the NHANES database for the relationship between blood trihalomethane concentrations and diabetes mellitus from 2013 to 2018.

In this cross-sectional study, data were collected from the 2013–2018 survey cycle in the NHANES database (*N* = 29,400). Participants aged no more than 60 years were excluded (*N* = 23,508). Meanwhile, there are no missing data for people with diabetes (*N* = 5,892). Subsequently, we removed the data with missing or zero weights (*N* = 280). In addition, we excluded missing data for trihalomethanes [including chloroform, bromodichloromethane, dibromochloromethane, bromoform, total trihalomethanes (TTHMs), and Br-THMs; *N* = 3101]. Finally, 2,511 participants were eligible for this study (**Figure 1**). Based on a population cross-sectional survey, the NHANES collects information on the health and nutrition of households in America. Each participant in the interview and evaluation provided informed consent. The National Health Statistics Research Ethics Review Board approved this biennial data collection. Likewise, the Centers for Disease Control and Prevention’s (CDC’s) Institutional Review Board also approved the protocol for the study.

### 2.2 Blood THM measurement

According to previous studies, the procedure for the determination of trihalomethanes is blood sampling by venipuncture, storing, determination, quality assurance (QA), and quality control (QC) (19, 20). Trihalomethanes are highly volatile and need to be stored in the room at 4°C. There are three methods for determining the concentration of trihalomethanes in blood, namely, solid-phase microextraction, gas chromatography, and mass spectrometry (21–23). BDCM, DBCM, and TBM were summed as Br-THMs, and TCM, BDCM, TBM, and DBCM were summed as TTHMs (13). In our research, values below the limit of detection (LOD) have been replaced by LOD/ √2 .

### 2.3 Determination of diabetes mellitus

According to the latest update of the diagnostic criteria for diabetes mellitus and the CDC’s undiagnosed diabetes definition (24), diabetes was defined as fasting plasma glucose (FPG) >7.0 mmol/L or 2 h postprandial >11.1 mmol/L in the oral glucose tolerance test (OGTT), or random blood glucose ≥11.1 mmol/L, HbA1C ≥ 6.5%, or self-reported. The diabetes population in this study was identified based on the above conditions’ diagnosis.

### 2.4 Definition of covariates

Factors that we considered may influence the relationship between blood trihalomethane concentrations and diabetes mellitus in diabetes as covariates. Information regarding patients’ age, sex, race, hyperlipidemia, marriage, poverty-to-income ratio (PIR), education, smoke status, body mass index, alcohol user status, and hypertension was collected. Ethnic groups include Mexican-Americans, non-Hispanic whites, non-Hispanic blacks, and other races. Marital information includes married/living with partner, widowed/divorced/separated, and never married. Educational level is categorized as below senior high school, senior high school, and above senior high school. BMI was calculated as weight/(height)^2^ kg/m^2^. The conditions for smokers include never smoked, used to smoke, and currently smoking. Alcohol user status was categorized as never drank, used to drink, low to moderate drinking (defined as ≤2 cups per day for women and ≤3 cups per day or ≤4 days of drinking per month for men), and heavy drinking (defined as ≥3 cups per day for women or ≥4 cups per day or ≥5 days of drinking per month for men). We defined hypertension as a systolic blood pressure (SBP) ≥130 mm Hg or diastolic blood pressure (DBP) ≥80 mm Hg or the presence of antihypertensive drug treatment or self-reported (Whelton et al., 2018). The PIR is divided into three levels, namely, PIR ≤ 1 (low income), PIR 1–3 (middle income), and PIR > 3 (high income). Participants were considered to have diabetes mellitus when they had a fasting glucose level >7 mmol/L, or a random glucose >11 mmol/L, or a glycated hemoglobin >6.5%, or 2 h postprandial >11.1 mmol/L in OGTT, or self-reported (24). The definition of hyperlipemia was triglyceride (TG) > 200 mg/dL, total cholesterol (TC) > 200 mg/dL, low-density lipoprotein (LDL) > 130 mg/dL, high-density lipoprotein (HDL)< 40 mg/dL (men)/50 mg/dL (women), or taking hypolipidemic drugs. Among the covariates, we found no statistically significant differences between diabetic and non-diabetic populations in age, marital, and smoking status (*p* > 0.05).

### 2.5 Statistical analysis

NHANES adopted a complex multi-stage probability sampling design; thus, appropriate weighting was used in our study. In our study, we described continuous variables (e.g., age) as mean ± standard deviation and categorical variables as percentages. In order to compare the differences among the groups, we used weighted ANOVA and chi-square test. Meanwhile, the multivariate logistic regression was conducted to explore the relationship between blood trihalomethane concentrations and diabetes mellitus. The older adults were assigned to quartile for TTHMs, whereas five groups were created for TCM (<75th, 75–87.5th, and ≥87.5th), BDCM (≤50th, 50–75th, and >75th), DBCM (≤25th, 25–75th, and >75th), TBM (≤25th, 25–75th, and >75th), and Br-THMs (<75th, 75–87.5th, and ≥87.5th). In order to reduce the influence of confounding factors on our result, we conducted model adjustments, Model 1: adjusted for age, race, and sex, and Model 2: adjusted for all covariates (age +sex +race + smoke+ alcohol user status + hypertension + marriage+ education + BMI + hyperlipidemia+ PIR). Subsequently, restricted cubic splines were used to further evaluate the blood concentration–response relationship between blood trihalomethane concentrations and diabetes mellitus, and the blood trihalomethane concentration–response curves were plotted clearly and show the relationship between blood trihalomethane concentrations and diabetes mellitus. To explore the contribution of trihalomethanes in reducing the incidence of diabetes, we used WQS. Finally, we also conducted a stratified analysis. Subgroups include race, sex, smoke, alcohol user status, marriage, education, PIR, hypertension, hyperlipidemia, and body mass index. The purpose was to explore the stability of the association between blood trihalomethane concentrations and diabetes mellitus in different subgroups. To explore multicollinearity in trihalomethanes, we plot a correlation heatmap. R-Version 4.21 is used to complete all our data analysis.

## 3 Results

### 3.1 Baseline information and correlation analysis

**Figure 2:**
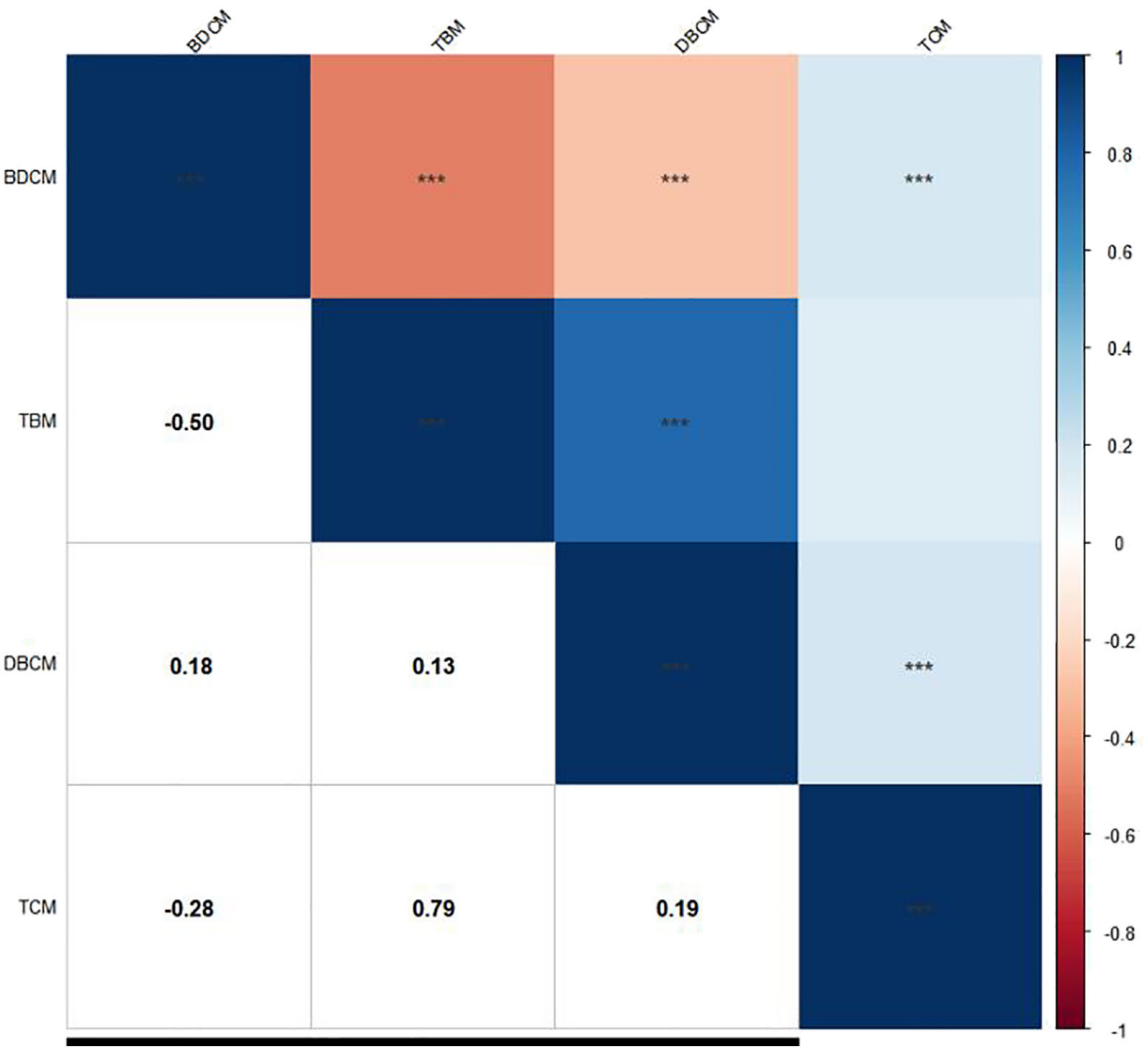
Exploring the correlation between trihalomethanes. ***means *P*<0.001.

The study had a total of 29,400 participants and ultimately included 2,511 members. The average age of most members is 69 years old (**Table 1**). In our study, the proportion of men and women was approximately identical. Over half of the participants had been educated in senior high school education or even obtained advanced degrees. Approximately 26.0% of participants had a high school diploma, and 15.7% of participants did not receive a high school education. Whether participants had diabetes or not, non-Hispanic whites make up most of our research group (75.7% of participants), most participants are low to moderate drinkers (67.5% of participants), most participants have hyperlipidemia (84.5% of participants), and most people suffer from hypertension (76.5% of participants). In addition, half of the participants had never smoked and more than half of those with diabetes had a body mass index above 30 kg/m^2^. We also found that the concentrations of TCM and BDCM in the blood of most participants were Q1, while others were Q2. In the correlation analysis (**Figure 2**), TBM and TCM have the strongest correlation (correlation coefficient: 0.79). The correlation coefficients between most trihalomethanes are less than 0.2.

**Table 1:** Baseline information, weighted, NHANES 2013–2018.

Characteristic	Trihalomethane			
	Overall	Non-diabetic	Diabetes	*p*
*N*	2,511	1,630	881	
**Age (years)**	69.88 ± 6.72	69.91 ± 6.77	69.84 ± 6.59	0.819
**GFR (mL/1.73 m^2^)**	102.02 ± 28.59	101.81 ± 29.15	102.4 ± 27.20	0.616
**Gender, *n* (%)**				0.003
Male	1,260 (45.9)	782 (43.1)	478 (52.6)	
Female	1,251 (54.1)	848 (56.9)	403 (47.4)	
**Race, *n* (%)**				<0.001
Mexican American	328 (4.6)	170 (3.5)	158 (7.4)	
Other races	556 (11.2)	345 (10.1)	221 (14.1)	
Non-Hispanic white	1,116 (75.8)	792 (78.8)	324 (68.3)	
Non-Hispanic blacks	501 (8.4)	323 (7.6)	178 (10.2)	
**Marriage, *n* (%)**				0.296
Married/living with partner	1,448 (62.5)	925 (63.0)	523 (61.3)	
Widowed/divorced/separated	900 (32.6)	602 (32.8)	298 (32.3)	
Never married	163 (4.9)	103 (4.3)	60 (6.4)	
**Education, *n* (%)**				<0.001
<High school	667 (15.7)	397 (14.3)	270 (19.3)	
High school	609 (26.0)	388 (25.3)	221 (27.6)	
>High school	1,235 (58.3)	845 (60.4)	390 (53.1)	
**Alcohol user status, *n* (%)**				0.01
Never	401 (12.3)	243 (10.2)	158 (17.5)	
Former	419 (14.0)	252 (13.2)	167 (16.0)	
Low to moderate	1,511 (67.5)	1,017 (70.2)	494 (61.1)	
Heavy	180 (6.1)	118 (6.4)	62 (5.4)	
**Smoke, *n* (%)**				0.456
Never	1,301 (51.8)	849 (51.9)	452 (51.6)	
Former	892 (38.6)	567 (38.2)	325 (39.5)	
Currently	318 (9.6)	214 (9.9)	104 (8.9)	
**BMI**, **kg/m^2^, *n* (%)**				<0.001
≤25 kg/m^2^	616 (22.5)	475 (26.7)	141 (12.3)	
25–30 kg/m^2^	942 (37.8)	637 (40.0)	305 (32.6)	
≥30 kg/m^2^	953 (39.7)	518 (33.4)	435 (55.1)	
**Hypertension, *n* (%)**				<0.001
Yes	1,998 (76.5)	1,247 (73.3)	751 (84.3)	
No	513 (23.5)	383 (26.7)	130 (15.7)	
**Hyperlipidemia, *n* (%)**				<0.001
Yes	2,102 (84.5)	1,323 (82.4)	779 (89.5)	
No	409 (15.5)	307 (17.6)	102 (10.5)	
**Chloroform, *n* (%)**				0.035
Q1 (<0.016 ng/mL)	1,867 (74.2)	1,185 (73.0)	682 (77.2)	
Q2 (0.016 to 0.024 ng/mL)	312 (12.9)	214 (13.1)	98 (12.3)	
Q3 (≥0.024 ng/mL)	332 (12.9)	231 (13.9)	101 (10.5)	
**Bromodichloromethane, *n* (%)**				<0.001
Q1 (≤0.004 ng/mL)	1,462 (57.4)	903 (55.3)	559 (62.8)	
Q2 (0.004 to 0.0042 ng/mL)	685 (27.4)	460 (27.6)	225 (26.7)	
Q3 (>0.0042 ng/mL)	364 (15.2)	267 (17.1)	97 (10.5)	
**Dibromochloromethane, *n* (%)**				0.084
Q1 (≤0.0035 ng/mL)	695 (28.5)	471 (29.0)	224 (27.1)	
Q2 (0.0035 to 0.0040 ng/mL)	1,511 (61.7)	955 (60.7)	556 (64.1)	
Q3 (>0.0040 ng/mL)	305 (9.8)	204 (10.2)	101 (8.9)	
**Bromoform, *n* (%)**				0.04
Q1 (≤0.0057 ng/mL)	743 (29.8)	510 (30.9)	233 (27.2)	
Q2 (0.0057 to 0.0060 ng/mL)	1,591 (64.2)	1,008 (63.3)	583 (66.4)	
Q3 (>0.0060 ng/mL)	177 (5.9)	112 (5.8)	65 (6.4)	
**Total trihalomethane, *n* (%)**				0.001
Q1 (<0.02 ng/mL)	332 (13.3)	215 (13.3)	117 (13.4)	
Q2 (0.02 to 0.028 ng/mL)	1,338 (53.7)	829 (52.4)	509 (56.8)	
Q3 (≥0.028 ng/mL)	841 (33.0)	586 (34.3)	255 (29.8)	
**Brominated trihalomethane, *n* (%)**				0.01
Q1 (<0.014 ng/mL)	627 (25.5)	422 (26.0)	205 (24.3)	
Q2 (0.014 to 0.018 ng/mL)	1,558 (62.0)	978 (60.8)	580 (64.9)	
Q3 (≥0.018 ng/mL)	326 (12.5)	230 (13.2)	96 (10.8)	

BMI, body mass index; GFR, glomerular filtration rate.

### 3.2 Association between blood trihalomethane concentrations and diabetes mellitus

**Figure 3:**
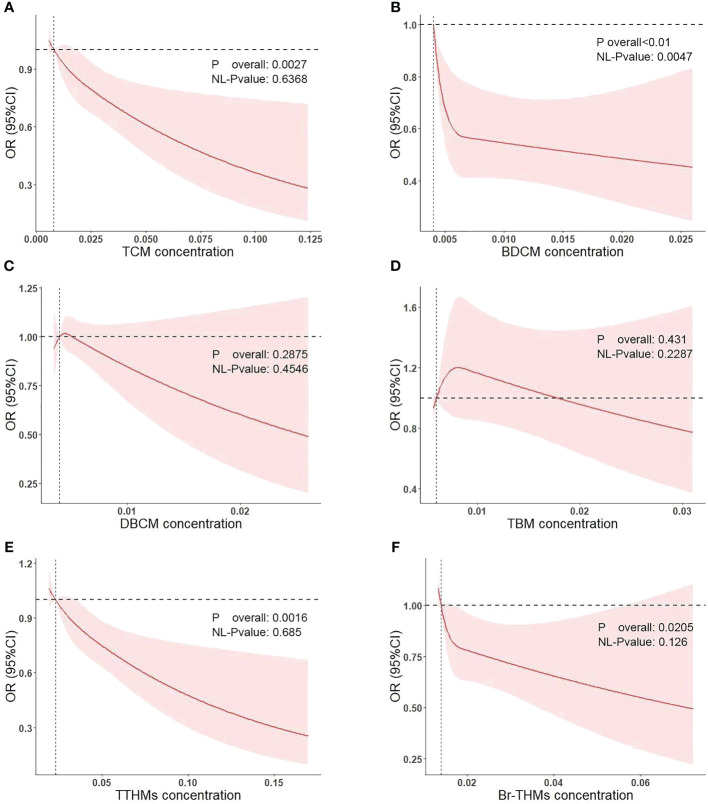
Association of diabetes mellitus with the TCM **(A)**, BDCM **(B)**, DBCM **(C)**, TBM **(D)**, TTHMs **(E)**, and Br-THMs **(F)** performed by restricted cubic spline analysis.

We conducted multivariate logistic regression analysis (**Table 2**). The results on blood levels of chloroform and diabetes mellitus are shown in model 1 (adjusted for covariates including age, sex, and race, OR = 0.71; 95% CI: 0.50–1.02; *p* = 0.068; *p* for trend = 0.094) and model 2 (adjusted for all covariates, OR = 0.68; 95% CI: 0.48–0.96; *p* = 0.029; *p* for trend = 0.061). For bromodichloromethane, the results are shown in model 1 (adjusted for covariates including age, OR = 0.54; 95% CI: 0.35–0.82; *p* = 0.005; *p* for trend = 0.002) and model 2(adjusted for all covariates, OR = 0.54; 95% CI: 0.35–0.82; *p* = 0.003; *p* for trend = 0.002). In the study of bromoform, dibromochloromethane, TTHMs, and Br-THMs, we did not find a statistically significant correlation with diabetes mellitus (*p* > 0.05). Meanwhile, we plotted restricted cubic spline curves to further evaluate the blood concentration–response relationship between blood trihalomethane concentrations and diabetes mellitus (**Figure 3**). Apart from the non-linear relationship in BDCM (*p* for nonlinear = 0.0047), we found a linear relationship in blood concentrations of all trihalomethanes. Moreover, blood chloroform, bromodichloromethane concentrations, and diabetes mellitus events in older adults in the US are negatively correlated (*p* overall = 0.0027; *p* overall< 0.01). Most members have lower concentrations of chloroform and bromodichloromethane in their blood than other types of trihalomethanes. Based on the results that we have obtained, we speculated that the protective effects of chloroform and bromodichloromethane on diabetes may be realized at low concentrations.

**Table 2:** Results of multiple logistic regression analysis of the association between trihalomethane and diabetes mellitus in older adults, weighted.

Chloroform	Model 1	*p*-value	Model 2	*p*-value
	OR (95% CI)		OR (95% CI)	
**Q1**	Ref	Ref	Ref	Ref
**Q2**	0.92 (0.62,1.36)	0.658	0.99 (0.66,1.50)	0.965
**Q3**	0.71 (0.50,1.02)	0.068	0.68 (0.48,0.96)	0.029
***p* for trend**	0.094		0.061	
Dibromochloromethane	OR (95% CI)	*p*-value	OR (95% CI)	*p* value
**Q1**	Ref	Ref	Ref	Ref
**Q2**	1.11 (0.88,1.39)	0.361	1.11 (0.89,1.39)	0.34
**Q3**	0.84 (0.54,1.29)	0.407	0.84 (0.55,1.28)	0.404
***p* for trend**	0.137		0.77	
Bromodichloromethane	OR (95% CI)	*p*-value	OR (95% CI)	*p*-value
**Q1**	Ref	Ref	Ref	Ref
**Q2**	0.87 (0.67,1.14)	0.305	0.89 (0.69,1.15)	0.374
**Q3**	0.54 (0.35,0.82)	0.005	0.54 (0.36,0.81)	0.003
***p* for trend**	0.002		0.002	
Bromoform	OR (95% CI)	*p*-value	OR (95% CI)	*p*-value
**Q1**	Ref	Ref	Ref	Ref
**Q2**	1.17 (0.92,1.48)	0.193	1.15 (0.91,1.46)	0.228
**Q3**	1.16 (0.60,2.24)	0.648	1.12 (0.59,2.11)	0.731
***p* for trend**	0.335		0.403	
Total trihalomethane	OR (95% CI)	*p*-value	OR (95% CI)	*p*-value
**Q1**	Ref	Ref	Ref	Ref
**Q2**	1.05 (0.71,1.55)	0.796	1.05 (0.72,1.53)	0.803
**Q3**	0.85 (0.55,1.33)	0.473	0.83 (0.54,1.27)	0.39
***p* for trend**	0.302		0.211	
Brominated trihalomethane	OR (95% CI)	*p*-value	OR (95% CI)	*p*-value
**Q1**	Ref	Ref	Ref	Ref
**Q2**	1.11 (0.83,1.49)	0.464	1.09 (0.81,1.47)	0.575
**Q3**	0.83 (0.51,1.35)	0.442	0.81 (0.50,1.32)	0.388
***p* for trend**	0.302		0.562	
**WQS (Negative)**			0.51 (0.37,0.7)	<0.001
**WQS (Positive)**			0.7 (0.43,1.13)	0.144

OR, odds ratio; 95% CI, 95% confidence interval. WQS, weighted quantile sum. Model 1: Adjusted for covariates (age, race, and sex). Model 2: Adjust for all confounding factors (age, sex, race, hyperlipidemia, marriage, poverty-to-income ratio, education, smoke status, body mass index, alcohol user status, and hypertension). Chloroform: Q1 (<0.016 ng/mL), Q2 (0.016 to 0.024 ng/mL), Q3 (≥0.024 ng/mL). Bromodichloromethane: Q1 (≤0.004 ng/mL), Q2 (0.004 to 0.0042 ng/mL), Q3 (>0.0042 ng/mL). Dibromochloromethane: Q1 (≤0.0035 ng/mL), Q2 (0.0035 to 0.0040 ng/mL), Q3 (>0.0040 ng/mL). Bromoform: Q1 (≤0.0057 ng/mL), Q2 (0.0057 to 0.0060 ng/mL), Q3 (>0.0060 ng/mL). Total trihalomethane: Q1 (<0.02 ng/mL), Q2 (0.02 to 0.028 ng/mL), Q3 (≥0.028 ng/mL). Brominated trihalomethane: Q1 (<0.014 ng/mL), Q2 (0.014 to 0.018 ng/mL), Q3 (≥0.018 ng/mL).

### 3.3 Trihalomethane exposure and diabetes mellitus in the WQS model

**Figure 4:**
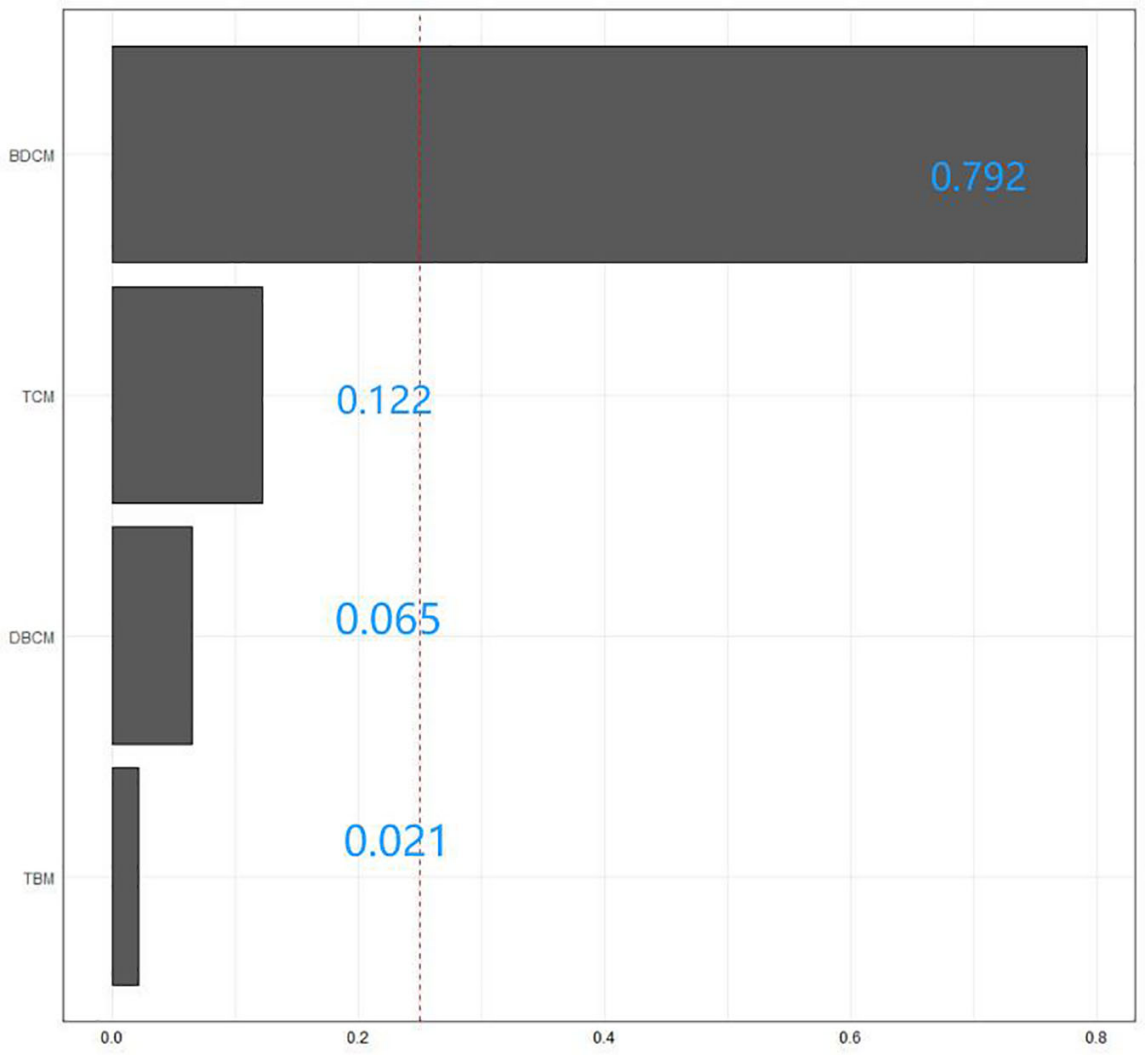
The WQS model weights of trihalomethanes on the prevalence of diabetes mellitus in the negative direction.

We used the WQS model to examine the relationship between the combined effects of these four tri-halomethanes and the incidence of diabetes. In terms of co-exposures, the WQS model found that trihalomethanes are inversely associated with diabetes (Model: OR = 0.51; 95% CI: 0.37–0.7; *p*< 0.001), with the top weight contributions from BDCM (79.2%) and TCM (12.2%). Positive WQS regression analysis showed no association between trihalomethanes and diabetes (Model: OR = 0.7; 95% CI: 0.43–1.13; *p* = 0.144), as shown in **Table 2** and **Figure 4**.

### 3.4 Subgroup analysis

Our purpose was to explore the stability of the association between blood trihalomethane concentrations and diabetes mellitus in different subgroups (**Tables 3**, **4**). In the results, we found that the *p* for interaction in most subgroups is higher than 0.1, indicating that the negative association between blood trihalomethane concentrations (chloroform and bromodichloromethane) and diabetes mellitus is robust. This means that these factors do not influence our results, such as gender, race, and education.

**Table 3:** Chloroform subgroup analysis.

Character	Q1	Q2	*p*	Q3	*p*	*p* for trend	*p* for interaction
**Sex**							0.449
Male	Ref	1.188 (0.574,2.462)	0.635	0.694 (0.415,1.163)	0.161	0.347	
Female	Ref	0.712 (0.393,1.288)	0.254	0.739 (0.457,1.193)	0.210	0.165	
**Race**							0.941
Mexican American	Ref	0.592 (0.177,1.982)	0.373	1.020 (0.393,2.646)	0.966	0.689	
Other races	Ref	1.043 (0.597,1.821)	0.879	0.776 (0.484,1.244)	0.283	0.321	
Non-Hispanic white	Ref	0.916 (0.546,1.539)	0.735	0.684 (0.430,1.087)	0.105	0.16	
Non-Hispanic blacks	Ref	0.742 (0.392,1.405)	0.348	0.648 (0.335,1.252)	0.189	0.152	
**Marriage**							0.078
Married/living with partner	Ref	1.216 (0.709,2.085)	0.469	0.648 (0.401,1.048)	0.076	0.224	
Widowed/divorced/separated	Ref	0.442 (0.224,0.871)	0.020	0.650 (0.366,1.154)	0.138	0.048	
Never married	Ref	1.353 (0.285,6.422)	0.695	1.923 (0.479,7.725)	0.345	0.322	
**Education**							0.372
<High school	Ref	0.615 (0.315,1.199)	0.149	0.634 (0.324,1.241)	0.178	0.119	
High school	Ref	1.470 (0.620,3.485)	0.373	1.109 (0.614,2.003)	0.727	0.477	
>High school	Ref	0.756 (0.373,1.532)	0.429	0.603 (0.332,1.093)	0.094	0.068	
**Smoke**							0.058
Never	Ref	0.620 (0.408,0.943)	0.026	0.560 (0.331,0.948)	0.032	0.016	
Former	Ref	1.116 (0.633,1.967)	0.698	1.008 (0.543,1.874)	0.978	0.873	
Now	Ref	2.602 (0.908,7.453)	0.074	0.816 (0.282,2.359)	0.700	0.778	
**Alcohol user status**							0.172
Never	Ref	0.794 (0.390,1.616)	0.516	0.455 (0.179,1.158)	0.096	0.081	
Former	Ref	1.009 (0.541,1.880)	0.978	0.501 (0.210,1.199)	0.116	0.166	
Low to moderate	Ref	0.852 (0.532,1.365)	0.497	0.720 (0.460,1.127)	0.147	0.124	
Heavy	Ref	2.707 (0.629,11.655)	0.173	2.777 (0.836, 9.226)	0.092	0.032	
BMI							0.229
≤25 kg/m^2^	Ref	0.787 (0.281,2.203)	0.641	0.937 (0.423,2.076)	0.871	0.676	
25–30 kg/m^2^	Ref	0.619 (0.322,1.192)	0.147	0.586 (0.370,0.928)	0.024	0.011	
≥30 kg/m^2^	Ref	1.502 (0.806,2.799)	0.194	0.823 (0.474,1.432)	0.483	0.919	
**Hyperlipidemia**							0.218
Yes	Ref	0.634 (0.190,2.114)	0.449	1.533 (0.540,4.352)	0.412	0.533	
No	Ref	0.884 (0.594,1.315)	0.534	0.627 (0.427,0.919)	0.018	0.02	
**PIR**							0.744
Low income	Ref	1.447 (0.662,3.161)	0.345	1.032 (0.475,2.244)	0.934	0.713	
Middle income	Ref	0.854 (0.589,1.240)	0.399	0.716 (0.416,1.234)	0.223	0.182	
High income	Ref	0.868 (0.430,1.751)	0.686	0.563 (0.295,1.076)	0.081	0.106	
**Hypertension**	Ref						0.956
Yes	Ref	0.783 (0.315,1.946)	0.591	0.693 (0.307,1.568)	0.370	0.361	
No	Ref	0.889 (0.603,1.312)	0.546	0.696 (0.476,1.018)	0.061	0.075	

Subgroup analyses were performed on the following covariates: age, sex, race, hyperlipidemia, marriage, poverty-to-income ratio, education, smoke status, body mass index, alcohol user status, and hypertension.

**Table 4:** Bromodichloromethane subgroup analysis.

Character	Q1	Q2	*p*	Q3	*p*	*p* for trend	*p* for interaction
**Sex**							0.823
Male	Ref	0.802 (0.524,1.230)	0.304	0.567 (0.310,1.036)	0.065	0.041	
Female	Ref	0.901 (0.641,1.268)	0.542	0.483 (0.256,0.910)	0.025	0.022	
**Race**							0.789
Mexican American	Ref	0.692 (0.346,1.385)	0.279	0.389 (0.173,0.872)	0.025	0.026	
Other races	Ref	0.999 (0.612,1.632)	0.998	0.772 (0.452,1.320)	0.335	0.413	
Non-Hispanic white	Ref	0.837 (0.578,1.212)	0.338	0.508 (0.289,0.891)	0.019	0.014	
Non-Hispanic blacks	Ref	1.088 (0.728,1.628)	0.670	0.589 (0.271,1.281)	0.175	0.239	
**Marriage**							0.61
Married/living with partner	Ref	0.797 (0.558,1.139)	0.207	0.613 (0.314,1.197)	0.147	0.097	
Widowed/divorced/separated	Ref	0.993 (0.633,1.557)	0.975	0.502 (0.262,0.961)	0.038	0.073	
Never married	Ref	0.772 (0.309,1.928)	0.569	0.211 (0.054,0.826)	0.027	0.061	
**Education**							0.745
<High school	Ref	1.118 (0.627,1.993)	0.700	0.781 (0.396,1.543)	0.468	0.844	
High school	Ref	0.746 (0.443,1.254)	0.261	0.429 (0.216,0.852)	0.017	0.012	
>High school	Ref	0.813 (0.571,1.157)	0.243	0.574 (0.274,1.199)	0.136	0.1	
**Smoke**							0.313
Never	Ref	0.957 (0.679,1.347)	0.795	0.413 (0.250,0.683)	<0.001	0.003	
Former	Ref	0.726 (0.441,1.195)	0.202	0.698 (0.383,1.274)	0.235	0.115	
Now	Ref	0.776 (0.310,1.943)	0.578	0.698 (0.321,1.518)	0.354	0.318	
**Alcohol user status**							0.276
Never	Ref	0.766 (0.458,1.281)	0.301	0.454 (0.151,1.358)	0.153	0.153	
Former	Ref	0.656 (0.316,1.362)	0.247	0.287 (0.154,0.535)	<0.001	0.007	
Low to moderate	Ref	0.784 (0.562,1.092)	0.146	0.570 (0.374,0.867)	0.010	0.006	
Heavy	Ref	1.931 (0.663,5.622)	0.217	1.554 (0.480,5.037)	0.448	0.305	
BMI							0.278
≤25 kg/m^2^	Ref	1.363 (0.803,2.311)	0.244	0.539 (0.230,1.266)	0.152	0.546	
25–30 kg/m^2^	Ref	0.717 (0.471,1.092)	0.118	0.417 (0.204,0.854)	0.018	0.01	
≥30 kg/m^2^	Ref	0.893 (0.626,1.275)	0.526	0.701 (0.395,1.246)	0.220	0.156	
**Hyperlipidemia**							0.55
Yes	Ref	0.697 (0.349,1.391)	0.297	0.363 (0.130,1.010)	0.052	0.05	
No	Ref	0.893 (0.674,1.183)	0.421	0.568 (0.395,0.817)	0.003	0.002	
**PIR**							0.402
Low income	Ref	1.112 (0.659,1.878)	0.683	0.804 (0.353,1.829)	0.594	0.727	
Middle income	Ref	0.782 (0.487,1.257)	0.303	0.363 (0.218,0.604)	<0.001	<0.001	
High income	Ref	0.837 (0.534,1.310)	0.427	0.689 (0.361,1.316)	0.252	0.217	
**Hypertension**	Ref						0.683
Yes	Ref	1.030 (0.500,2.123)	0.934	0.784 (0.300,2.050)	0.612	0.72	
No	Ref	0.821 (0.584,1.154)	0.248	0.487 (0.309,0.767)	0.003	<0.001	

Subgroup analyses were performed on the following covariates: age, sex, race, hyperlipidemia, marriage, poverty-to-income ratio, education, smoke status, body mass index, alcohol user status, and hypertension.

## 4 Discussion

This cross-sectional analysis of older adults in the US showed that blood BDCM and TCM concentrations were associated with diabetes mellitus. Furthermore, we believe that this relationship is negatively correlated, indicating that BDCM and TCM may play a protective role in diabetes mellitus development. These associations, however, were not observed in other trihalomethanes (DBCM, TTHMs, TBM, and Br-THMs). Most members had lower concentrations of chloroform and bromodichloromethane in their blood than other types of trihalomethanes. We speculate that the protective effects of chloroform and bromodichloromethane on diabetes may be realized at low concentrations.

Previous population studies have shown that all trihalomethanes, with the exception of TBMs, have protective effects in the diabetic population (18), which seems to contradict our conclusions. In fact, our study does not deny this conclusion. It is well known that the pathogenesis of diabetes mellitus is associated with abnormalities in immune system and metabolism. Metabolic abnormalities, such as insulin resistance, are critical in the development of diabetes mellitus. The immune system and metabolic function are often two-way linked. On the one hand, inflammation can promote metabolic abnormalities such as obesity and diabetes. On the other hand, the metabolic factors, in turn, may regulate immune cell function (25). Our study was conducted in older adults in the US of the diabetic population. We considered that older adults might be a representative group with immunometabolism disorders. Older adults usually have a low level of immune system and tend to show chronic low-grade inflammation, which is related to the pathogenesis of many age-related diseases (atherosclerosis, Alzheimer’s disease, osteoporosis, and diabetes) (26). In addition, it has been reported that trihalomethanes also play a role in the development of asthma and other diseases (27). It is well known that the pathogenicity of trihalomethanes is inevitable. Therefore, we consider that studying lower concentrations of trihalomethanes is more meaningful in exploring their role for diabetes mellitus. Compared with the younger group with good body management and love of sports, the living habits of older adults are usually reducing physical activity (PA) and exercise, which means less exposure. In 2006, the standard for DBPs in water has been revised by the United States Environmental Protection Agency (28). Compared to Rieder’s study of NHANES from the 1999–2006 survey cycle, the information for the 2013–2018 survey cycle was collected according to the new standards. The World Health Organization’s guidelines on drinking water also require limiting the concentration of trihalomethanes in daily water (WHO G, (29)). Unfortunately, we have not explored the specific THM concentration that can exhibit antidiabetic activity without causing other diseases, which is the direction of our next research.

According to previous studies, the chloroform fraction of plants (such as *A. vogelii* Planch root bark) can exhibit antidiabetic activity in rats with diet- and alloxan-induced obesity–diabetes (14), which may be related to the extracted components of chloroform fraction, including quebrachitol (QCT), loganin, sweroside, oleoside 11methyl ester, and ferulic acid (30). QCT can act as a β-glucosidase inhibitor and thus resist diabetes (31). Sweroside can the regulation of phosphoenolpyruvate carboxykinase gene expression and then mimic insulin to resist diabetes (32). Loganin, oleoside 11-methyl, and ferulic acid ameliorates hyperglycemia by reducing oxidative stress levels (33–35). Most components are associated with oxidative stress levels. This is robust evidence that chloroform may be a protective factor for diabetes. Unfortunately, no extracts of BDCM have been reported for their antidiabetic effects in animal models of diabetes. Our research can be supplementary to the antidiabetic effects of chloroform and it can also stimulate the development of animal models to validate the antidiabetic effects of BDCM.

In addition, some studies have concluded that Br-THMs are a risk factor for diabetes (15, 16). We believe that their study may have some limitations that lead to the opposite conclusion from ours. Firstly, the study is not in a larger sample size population to validate the accuracy of conclusion. Secondly, based on multivariate logistic regression models for T2DM, we found that none of their trihalomethane-related data was statistically significant (*p* > 0.05). However, we also agree with another view that exposure of Br-THM can modulate leptin and insulin sensitivity; thus, we do not completely reject the conclusions of these two studies. Further experiments are still needed to demonstrate the complex mechanisms.

Finally, our study inevitably has some limitations. First, we cannot avoid the shortcomings of reverse causality in cross-sectional studies. Second, we may also be affected by non-measured or uncontrolled covariates. Third, because the intake of trihalomethanes causes various systemic diseases, we were not able to provide a definite concentration as an indicator of anti-diabetes. Fourth, we do not consider the impact of living habits on data measurement. For example, some people love swimming while others do not. Finally, although NHANES measured a large amount of data on trihalomethanes, we did not explore the accuracy of our conclusions in the context of co-exposure to other DBPs, and the steady-state exposures of different DBPs may be inconsistent; thus, it is not possible to control for co-exposure under the same variable. Meanwhile, for older adults, it is still worth considering whether it is necessary to increase water-use activities to increase the concentration of trihalomethanes in the blood. These activities (such as swimming and sauna) are strongly related to blood THM concentrations in the older adults, which means that they may also experience other diseases. Investigating the antidiabetic effects of trihalomethanes at specified levels in the context of not causing other diseases is a new research direction, which is indicated by our study.

## 5 Conclusions

Our study demonstrated a negative correlation between blood concentrations of chloroform and bromodichloromethane and the incidence of diabetes in older adults in the US, which indicates that they may reduce the incidence of diabetes. Compared to other trihalomethanes, chloroform and bromodichloromethane concentrations in older adults in the US are lower. This greatly reduces the potential for other diseases. The pathogenicity of trihalomethanes is not negligible, and our study provides a new reference for standards for chlorinating water for disinfection, but the necessity of increased water use activities in older adults in the US is worth considering. Investigating the antidiabetic effects of specific levels of trihalomethanes without causing other diseases is a new direction for research.

## Data availability statement

Publicly available datasets were analyzed in this study. This data can be found here: https://www.cdc.gov/nchs/nhanes/index.htm.

## Ethics statement

The studies involving humans were approved by NCHS Research Ethics Review Board. The studies were conducted in accordance with the local legislation and institutional requirements. The participants provided their written informed consent to participate in this study.

## Author contributions

TC: Writing – review & editing, Writing – original draft, Investigation, Data curation. HH: Writing – review & editing, Investigation. WT: Writing – review & editing, Data curation. HZ: Writing – review & editing, Supervision, Resources, Project administration, Funding acquisition. ZL: Writing – review & editing, Validation, Software, Methodology, Formal analysis, Conceptualization.
